# The Impact of MNRI Therapy on the Levels of Neurotransmitters Associated with Inflammatory Processes

**DOI:** 10.3390/ijms21041358

**Published:** 2020-02-18

**Authors:** Tatiana V. Tatarinova, Trina Deiss, Lorri Franckle, Susan Beaven, Jeffrey Davis

**Affiliations:** 1 Department of Biology, University of La Verne, La Verne, CA 91750, USA; 2 Functional Genomics Group, Vavilov Institute for General Genetics, Moscow 119991, Russia; 3 Forest Genomics Laboratory, Siberian Federal University, Krasnoyarsk 660041, Russia; 4 Bioinformatics Center, Information Transmission Problems Institute, Moscow 127051, Russia; 5 Research Department, United1Front Foundation, Minneapolis, MN 55111, USA; deisstrina@gmail.com; 6 Laser Health Department, Laser Health, Orlando, FL 33709, USA; lorrifranckle@gmail.com; 7 Family Medicine, St. Petersburg Free Clinic, St. Petersburg, FL 33701, USA; scbeaven@gmail.com; 8 Family Medicine, Prairie Health and Wellness, Wichita, KS 67206, USA; jeff@prairiehealthwellness.com

**Keywords:** neurotransmitters, inflammation, MNRI

## Abstract

The neurotransmitter levels of representatives from five different diagnosis groups were tested before and after participation in the MNRI^®^—Masgutova Neurosensorimotor Reflex Intervention. The purpose of this study was to ascertain neurological impact on (1) Developmental disorders, (2) Anxiety disorders/OCD (Obsessive Compulsive Disorder), PTSD (Post-Traumatic Stress disorder), (3) Palsy/Seizure disorders, (4) ADD/ADHD (Attention Deficit Disorder/Attention Deficit Disorder Hyperactive Disorder), and (5) ASD (Autism Spectrum Disorder) disorders. Each participant had a form of neurological dysregulation and typical symptoms respective to their diagnosis. These diagnoses have a severe negative impact on the quality of life, immunity, stress coping, cognitive skills, and social assimilation. This study showed a trend towards optimization and normalization of neurological and immunological functioning, thus supporting the claim that the MNRI method is an effective non-pharmacological neuromodulation treatment of neurological disorders. The effects of MNRI on inflammation have not yet been assessed. The resulting post-MNRI changes in participants’ neurotransmitters show significant adjustments in the regulation of the neurotransmitter resulting in being calmer, a decrease of hypervigilance, an increase in stress resilience, behavioral and emotional regulation improvements, a more positive emotional state, and greater control of cognitive processes. In this paper, we demonstrate that the MNRI approach is an intervention that reduces inflammation. It is also likely to reduce oxidative stress and encourage homeostasis of excitatory neurotransmitters. MNRI may facilitate neurodevelopment, build stress resiliency, neuroplasticity, and optimal learning opportunity. There have been no reported side effects of MNRI treatments.

## 1. Introduction

**Figure 1:**
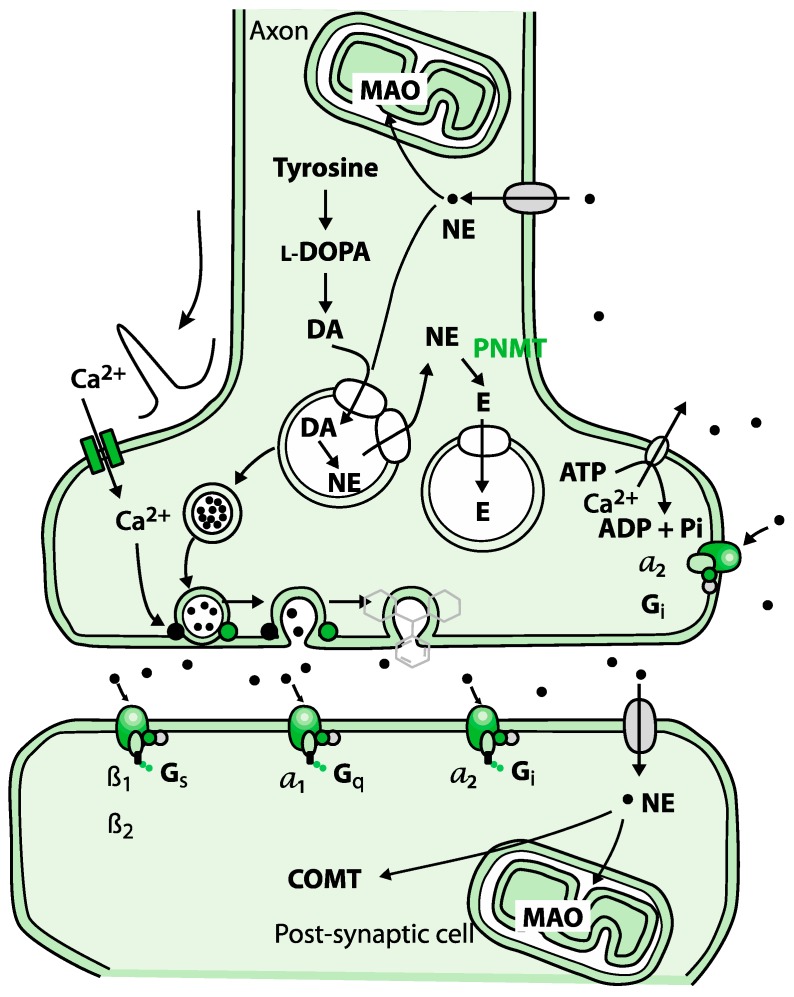
Biosynthesis of adrenaline involves a series of enzymatic reactions.

Healthy regulation and balance of the neurotransmitters are essential to the homeostasis of the body since chronic neurodegenerative diseases often have mitochondrial and neuroinflammation dysfunctions. Inflammation processes are manifested in many diseases and disorders such as cerebral palsy, epilepsy, autism, post-traumatic stress disorder (PTSD), as well as autoimmune diseases [1,2,3]. The elevation of cortisol in these conditions becomes the driving factor for increased levels of phenylethanolamine N-methyltransferase (PMNT). This effect is demonstrated by a reduction in epinephrine levels before a change in norepinephrine, thus leading to oxidative stress or inflammation [4,5]. Another critical enzyme is monoamine oxidase A (MAO), mediating the turnover of noradrenaline. MAOs are needed for the breakdown of monoamines ingested in food and serve to inactivate monoamine neurotransmitters. MAO is correlated with neurotransmitter excitation, and elevated levels of MAO are found in the same disorders as PMNT. MAO assists with the degradation of aspects of the serotonergic, adrenergic, and dopaminergic systems; therefore, MAO dysfunction has been detected in ADHD and ASD cases. The third factor is the N-methyl-D-aspartate receptor (NMDA), which is a glutamate receptor (Figure 1). Excitotoxicity caused by overactivation of this receptor has been connected to many neurogenerative disorders, especially epilepsy [6,7,8,9].

Adrenaline can produce retrograde enhancement of long-term memory in humans. The release of adrenaline under emotional stress can modulate memory consolidation of the events, ensuring memory strength that is proportional to memory importance.

When dysfunction of these enzymes and receptors occurs, then the symptomatic issues become more pronounced and create chronic inflammation, thus driving diseases and disorders further into pathology [10]. In our study, the MNRI approach trended towards normalization of PMNT, MAO, and NMDA functions effectively and non-pharmaceutically without adverse effects, thereby mitigating disease processes.

This study intends to determine the effectiveness of the MNRI therapy for disorders such as (1) developmental disorders, (2) anxiety disorders, (3) palsy/seizure disorders (4) ADD/ADHD, and (5) ASD disorders utilizing analysis of neurotransmitter markers. The assessment of neurotransmitters via urinalysis is both the least invasive and most optimal way to measure these biomarkers since venipuncture and cerebral spinal fluid collection methods have various complications [11,12,13]. Importantly, the optimal range for CSF neurotransmitter has yet to be established.

Utilization of urine using Fluorometric methods and High-Performance Liquid Chromatography (HPLC) methodology have expanded parameters to greater specificity and sensitivity, allowing an even more comprehensive range of clinical applications [14]. There are several advantages to using this approach:

A. Reliability and quality assurance are achieved using the CLIA certified labs.
B. Since the 1960s, several studies depended upon biomarkers of neurotransmitters and metabolites [14].
C. Several studies concluded that there is a direct correlation between urine and CNS neurotransmitters [15,16,17,18]. Many researchers and clinicians are formally using urinary neurotransmitters as biomarkers and as a diagnostic and assessment tool [15,17,18,19].

The following neurotransmitters were evaluated in this paper: (1) epinephrine, (2) norepinephrine, (3) dopamine, (4) serotonin, (5) glutamate, (6) glycine, (7) histamine, (8) DOPAC, and (9) 5HIAA. These chemical transmitters function throughout the whole human organism and communicate by targeting specific cells. The immune and central nervous systems are interconnected and co-responsive with each other. A complex modulatory system exists between the central nervous (CNS) and immune systems. A body is hardwired to have an inverse relationship between the sympathetic (SNS) and parasympathetic (PNS) nervous systems. Neuroendocrine hormones regulate cytokines balance; the immune system utilizes inflammatory, anti-inflammatory, autoimmune, inflammasome loops that work simultaneously to ensure homeostasis.

These systems allow for ongoing adaptations on a cellular, psychological, immunological, and structural level. Oxidative stress can facilitate the infraction of homeostasis between immune and nervous systems [20,21,22,23,24].

Norepinephrine and epinephrine affect alpha and beta receptors. These are part of the fight-and-flight responses that are initiated under stress. Norepinephrine is a precursor to epinephrine. The first response is norepinephrine through the sympathetic nervous system. Activation of norepinephrine raises alertness, preparing the body for action by increasing heart rate and blood pressure, mobilizing glucose, and facilitating blood flow to skeletal muscles. If the stress continues or increases, norepinephrine is converted to epinephrine [17,25,26,27].

Norepinephrine can be activated by both pre- and post-synaptic adrenergic receptors, thereby, it can be either released in the locus coeruleus, where it is beneficial for long-term memory as a neurotransmitter, or in the adrenal medulla, where it functions as a hormone.

**Figure 2:**
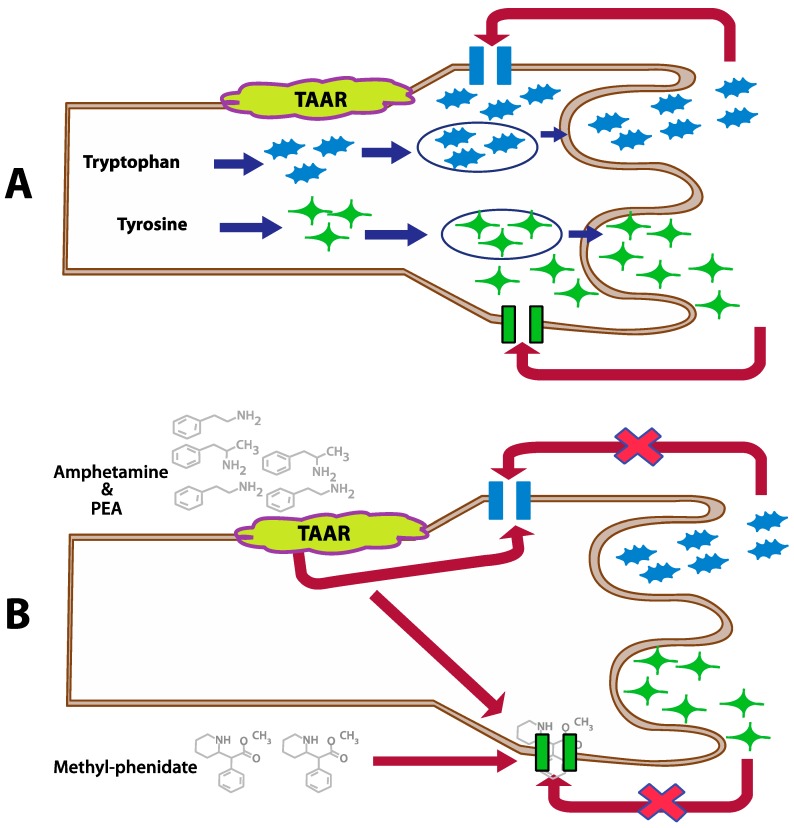
Inhibition of the dopamine and serotonin transporters by PEA, amphetamine, and methylphenidate in case of a normal action (**A**) and modulated by PEA and amphetamine (**B**) (This figure is redrawn from [25]).

Dopamine is a hormone, as well as a neurotransmitter. Dopamine is a precursor in the synthesis of the neurotransmitters norepinephrine and epinephrine. There are two common functions of dopamine: (1) in association with the motivational construct of reward-motivational behavior, and (2) in motor control. Dopamine function is directly associated with the kidneys, pancreas, intestinal mucosa, digestion, and gastrointestinal motility (Figure 2) [16,28,29].

Glycine works as an immunomodulator, a cytoprotective agent, and an anti-inflammatory regulator. The overall health of a person directly depends on the functioning of glycine. Whether it is produced in the liver for detoxification, or it is facilitating the synthesis of bile and amino acids, it plays a role in suppression of activation of transcription factors and the formation of free radicals and cytokines. Glycine also participates in building DNA and RNA molecules. Excessive amounts of glycine can lead to decreased energy, anxiousness, sleep difficulties, as well as immune dysregulation and digestive stress [30].

**Figure 3:**
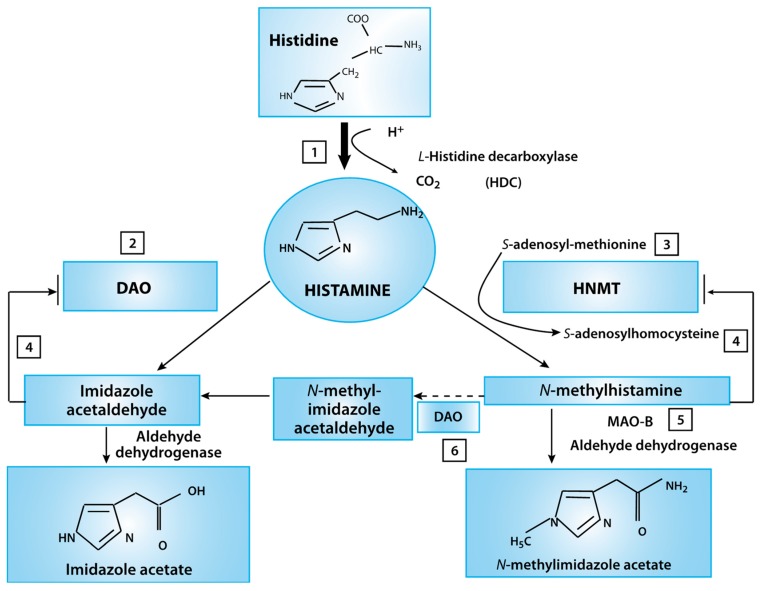
Histamine metabolism diagram. Oxidative deamination (by diamine oxidase–DAO) or intracellular methylation (by histamine-N-methyltransferase HNMT) are two possible pathways. If the activity of any of these enzymes (DAO or HNMT) is insufficient due to inhibition by reaction products, histamine is accumulated (This figure was re-drawn from [29]).

Histamine, which is well-known for its inflammatory response, is also involved in the regulation of the overall physiological responses of the gut and local immune response (Figure 3). The posterior hypothalamus houses the tuberomammillary nucleus (TMN), where the histamine neurons are responsible for the arousal portion of the sleep-wake cycle. The locus coeruleus neurons fire most rapidly during wakefulness; firing is slower during rest and completely stops during the REM sleep stage [31].

DOPAC is a metabolite of dopamine. Dysfunction of dopamine leads to various nervous system diseases or disorders. If the origination of dysfunction is oxidative stress, it will have a direct impact on health. MAO, associated with oxidative stress, is the enzyme that breaks down dopamine to DOPAC [29,32,33].

## 2. Results

Our study (Table 1, Table 2, Table 3, Table 4 and Table 5) shows that MNRI 8-day intensive treatment experience causes changes in the neurotransmitter activity of the patients.

In the ***developmental disorders*** study group (Table 1), we found a significant reduction in epinephrine (*p*-value < 0.05, the medium effect of −0.5), but no change in the norepinephrine level. In the ***anxiety*** study group (Table 2) we observed reduction in glutamate and glycine (small effects of −0.3 and −0.2, respectively). ***Palsy and seizures*** study group (Table 3) showed several notable changes that may indicate the reduction of inflammatory or oxidative stress. Levels of four neurotransmitters (epinephrine, norepinephrine, glycine, and GABA) demonstrated significant changes. Palsy and seizure patients can complain about ataxia, rigidity, athetosis, eating disorders, spasticity or hypo/hyperactive muscle control [34]. Epidemiology of seizure disorders are not fully understood; however, a generally accepted definition is that of a heterogeneous compilation of various syndromes or neurological conditions that present with recurrent, unprovoked, paroxysmal seizures [35].

An analysis of the findings allows us to conclude that MNRI consistently addresses symptomatic results indicative of neurotransmitter imbalances. These imbalances often present with mood disorders, cognitive impairment, focus difficulty, immune disorders, behavior, and social assimilation issues, physiological disorders such as spasticity and development, and digestive disorders, all of which are present in each of these diagnostic groups. The results, along with collaborative statements from caregivers, give compelling information in the roll of MNRI and the neurological homeostasis of the body. Many participants with the disorders presented utilize medications that bring multiple side effects or invasive interventions that carry risks and additional stressors. Our results demonstrate that in as little as eight days, a trend in change towards homeostasis occurs. This outcome is achieved by the self-regulation of PMNT and MAO, facilitating neurodevelopment and reduction of oxidative stress.

Neurotransmitters are intricately involved in homeostasis; they are crucial to modulating behaviors and functioning of the immune system. These chemical messages are transmitted by neural synapses specific to each transmitter; a neurotransmitter is released by a presynaptic neuron and then acts on a postsynaptic target cell, and every neurotransmitter has multiple receptor molecule types. Neurotransmitters differ in their mechanism of action regarding action potential: they can be either excitatory or inhibitory. Inhibitory will prevent action potential, whereas excitatory will enhance action potential. Several studies suggested [33,36,37,38] that neurotransmitters have respective and collaborative involvement in cognitive processes, including memory. The neurochemical imbalance affects spontaneous decision making [38].

Several studies supported the existence of intricate communication between the immune system and the nervous system [39]. It has been demonstrated that in addition to neurotransmitters signaling through lymphocyte cell-surface receptors initiating modulation, leukocytes can release neurotransmitters achieving autocrine and paracrine modulation [3].

## 3. Discussion

We limit our discussion to those neurotransmitters that were significantly affected by the treatment (with *p*-values < 0.05). Comparisons include control (Ct) vs. pre-treatment (Pr), control vs. post-treatment (Ps), and pre- vs. post-treatment. Below, we discuss our findings for different groups of patients.

### 3.1. Developmental Disorders

Implications of chronic stress levels leading to inflammation (existence of stress-related diseases) were demonstrated in many studies. A persistent increase in allostasis can lead to pathophysiology [3]. A study conducted at Uppsala University showed that the more serotonin their subjects produced, the more anxious they became [40]. In our research, the *developmental disorders* group had high levels of serotonin pre-test and achieved a reduction of 10% post-treatment.

There is a relationship between the levels of epinephrine and norepinephrine. Norepinephrine is continually released into the bloodstream, while epinephrine is synthesized from norepinephrine only under stress. Because there was no change in norepinephrine, we speculate that enzyme phenylethanolamine N-methyltransferase (responsible for conversion of norepinephrine to epinephrine) is operating at a reduced capacity, suggesting a reduction in cortisol levels. Consequently, less inflammatory stress will result in diminished anxiety, a better coping mechanism, and the ability to reach higher cognitive functioning.

Reduction in serotonin along with unchanged levels of its byproduct 5HIAA may lead to reduced anxiety [41,42]. Improvement in symptoms was collaborated by caregivers and participants: less stress, anxiety, and improved sleep patterns. Explicitly, parents reported that children who received MNRI treatment begin to have freedom of speech and improvement in physical development, and better muscle control and bowel elimination.

High levels of glycine are indicative of potential neurological disorders [43]. Post-treatment, the glycine levels were closer to the normal range, while remaining above the healthy control (Table 1).

When more than 0.5 g/kg of glycine is absorbed, adverse central nervous reactions occur in children [43]. An excessive amount of glycine will result in anxiousness, sleep disturbances, and immune and digestive dysregulation. On the other hand, glycine is a well-known cytoprotective agent playing a role in immunomodulation. It acts on inflammatory cells such as macrophages to suppress the activation of transcription factors and the formation of free radicals and inflammatory cytokines [44]. In the long term, according to parents and caregivers, the treatment resulted in less frequent hospitalizations, improved respiratory systems (fewer interventions or medications), and less medication was required to support digestive problems.

### 3.2. Anxiety/OCD (Obsessive Compulsive Disorder)/PTSD (Post-Traumatic Stress Disorder)

Symptomatic characterizations of this study group are anxiety, depression, sleep disturbances, immunity issues, rage, headaches, heart palpations, and panic attacks. All these symptoms make a body susceptible to immune disorders. New studies in neuro-psycho-pharmacological have demonstrated connections between immune disorders and neuropsychiatric diseases [45]. Interruptions in the excitatory inflammation process will translate into less symptomatic responses and the ability to enjoy life and having adaptive coping skills.

We observed a simultaneous decrease of GABA and glutamate levels; therefore, the reduction of glutamate may cause a reduction of GABA. GABA has inhibitory effects on the CNS; it also works with t-cells [45]. Since we observed an increase in DOPAC and no change in dopamine, norepinephrine, or epinephrine levels, MAO activity is likely to be enhanced after the MNRI treatments. Pharmaceutical MAO inhibitors are commonly used; however, there are multiple side effects and tolerance build-up. Our study showed MAO enhancement without reported side effects.

Participants reported being more relaxed, to have less repetitive or negative thoughts. Participants reported a reduced need for medication, increased independence from caregivers. Fewer outbursts and increased self-control were also reported. Several participants who have undergone more than one MNRI conference were able to resume normal daily activities and achieved significant independence from caregivers.

### 3.3. Palsy and Seizures

The level of glycine (which is a marker for inflammation) showed a small reduction (−0.03) while remaining about the control range post-treatment. While proper functioning glycine is a protective agent, its excess has been found to exasperate symptoms of palsy and seizures. Nonketotic hyperglycinemia (NKH) is one example of this. NKH presents within the first week of birth with low muscle tone, need for ventilation, weakness, and possible seizures. Abnormal jerking movements and hypotonia of all systems were also reported [46]. Glycine is present in both the brainstem and spinal cord and forebrain, involved in various motor and sensory functions. Forebrain studies have concluded that the N-methyl-D-aspartate (NMDA) receptor-gated ion channel is regulated by glycine [47]. NMDA is a glutamate receptor, modifying the action of glycine [48], so glycine can switch between being excitatory and inhibitory. The participants reported a reduction in the frequency of seizures, spasticity, or hypotonic symptoms. Multiple MNRI conferences lead to a reduction in the need for surgical interventions.

Our study demonstrated a small reduction in epinephrine (small effect -0.3) and an elevation in norepinephrine (small effect 0.3). These observations, combined with no change in dopamine, imply that the MNRI treatment did not change dopamine levels, acting directly on norepinephrine and epinephrine. Because epinephrine levels decrease, and epinephrine levels increase without dopamine involvement, this may indicate the involvement of cortisol or PMNT, leading to a reduction in oxidative or inflammatory stress.

The reduction of glycine and GABA can also impact the respiratory system (significantly reduced with *p*-value < 0.05, medium effect −0.2). GABA inhibits the CNS; several studies found increased tonic inhibition related to extrasynaptic GABA-A receptors in traumatic brain injuries or strokes, contributing to subsequent functional impairment [49]. High levels of GABA can have adverse effects on patients, such as gastric distress, constipation, fatigue, breathing difficulties, muscle weakness, and reduced appetite. The reduction of the level of GABA brings alleviation of these symptoms.

### 3.4. Attention Deficit Disorder/Attention Deficit Disorder Hyperactive Disorder (ADD/ADHD)

Patients diagnosed with these disorders suffer from impulsivity and inattention. Cytokines have an intricate role in tryptophan metabolism and dopaminergic pathways. Therefore, a change in the levels of anti-inflammatory and pro-inflammatory cytokines may influence the pathogenesis of ADHD [49,50]. Individuals who have ADD or ADHD are known to have a highly efficient and more dopamine reuptake inhibitors. If dopamine level decreases too quickly, it is no longer able to fulfill its role. Therefore, stimulants are utilized to block dopamine transporters, thus allowing dopamine more time to impact cells.

Table 4 shows results for the control and treatment groups. There is a small (−0.3 effect, *p*-value < 0.05) change of dopamine and an elevated DOPAC (*p*-value < 0.01, the medium effect of 0.6), suggesting that there is an increase in the MAO activity. Therefore, MNRI treatment may be impacting MAO activity.

Microglia are the principal resident immune cells of the brain. When activated, microglial cells release pro-inflammatory cytokines and other factors such as glutamate, contributing to neuroinflammation. Crosstalk between peripheral immune cells and microglia can potentiate inflammation both in the periphery and in the brain. Post-MNRE levels of glutamate and histamine are lower compared to the pre-MNRE levels (glutamate is lowered by −0.4, *p*-value < 0.05, histamine at *p*-value < 0.05, effect −0.5). Recall that glutamate, glycine, and histamine are inflammatory markers, and the histamine is a substrate of MAO. Therefore, it is possible that MNRI not only reduces the inflammation but also assists in the modulation of MAO activity in the body. Naturally impacting reductions of these neurotransmitters may allow participants to have fewer incidents of behavior related issues at school, and social settings and improved learning capacity.

### 3.5. Autism Spectrum Disorders

Patients with ASD frequently have high levels of oxidative stress and report extremes of hyper- or hypo-arousal. We have observed (Table 5) no change in dopamine or epinephrine levels; however, there was a reduction in norepinephrine at *p*-value <0.01 and the small effect of −0.4. Monoamine oxidases (MAO) metabolizes norepinephrine. Both too much and too little of MAO negatively affects its function. This imbalance is associated with depression, attention deficit, addictions, migraines, and irregular sexual maturation. Serotonin is another MOA target; the treatment reduced the serotonin level (*p* < 0.05 with a small effect of −0.3). MAO is responsible for metabolizing serotonin to 5HIAA. Prior studies showed that there is an impairment in the proper functioning of MAO in subjects with Autism and ASD sub-diagnosed [51]. Decreased activity of MAOs may result in elevated levels of monoaminergic neurotransmitters, such as serotonin, which plays a critical role in autism [52]. Caregivers stated that after the MNRI treatment, the participants had less anxiety, they slept better and recognized social cues. Therefore, we suggest that MNRI may activate modulation of MAO. Even though palmitoylethanolamide (PEA) is sometimes used to treat ASD patients, its excess is associated with “mind racing,” sleep disturbances, anxiety, irritability, and even schizophrenia [53].

## 4. Materials and Methods

### 4.1. MNRI Method

The MNRI method was initially developed in Russia in 1989 and further developed in Eastern Europe over the subsequent years. Several scientific studies and clinical observations have shown that this non-pharmacological treatment modality was significant positive results towards improvement in the neurological functioning in individuals with sensorimotor or reflex development deficits, behavior disorders, speech and language pathologies, and learning disabilities [54,55,56,57,58,59,60]. MNRI appeared in the USA in 1996 and has gradually been accepted by professionals in over 40 countries. MNRI is a neuromodulation method as it facilitates the neurodevelopment in individuals with various neurological deficits and enables them to improve their reflex circuit functions—integration of their sensory and motor aspects, postural control, motor coordination, and physiological markers [57,58]. This neuroplasticity and skill development enable improved functioning, development, and learning [18,54,55,56,57,58,59,60]. The MNRI therapy program is based on the theory that impaired reflex circuits can be reconstructed and re-integrated, which involves awakening the sensorimotor genetic memory in individuals even with severe diagnosis (such as CP and brain damage) [57,58]. MNRI is an evidence- and research-based therapeutic program which utilizes neuroplasticity via reflex integration techniques for the neuro-sensorimotor-cognitive development of children and adults with neuro deficits and learning problems [61,62]. Over the past 30 years, numerous studies were conducted and many articles demonstrating the efficiency of the method were published by Dr. S. Masgutova and her scientific colleagues from various international institutions.

Exercise-based program MNRI is based on the technique called “repatterning” which essentially means re-educating, recoding, rerouting, and paving the reflex nerve pathways specific for dynamic and postural reflex schemes (e.g., Babinski, Automatic Gait, Bauer Crawling, Hands Grasp, and others) [61]. The stimulation of reflex pathways is aimed at strengthening and stabilizing the traces of genetic sensory-motor memory and at the activation of the innate defense mechanisms through the body’s neuroendocrine hormonal ‘alarm’ system (HPA axis) (hypothalamus-pituitary-adrenal gland stress response cycle activation) in times of stress or danger [20]. MNRI exercises stimulate innate neuro-regulatory mechanisms and enhance stress resiliency immune function [55,56,60]. Repatterning activates the extrapyramidal nervous system (peripheral nerves, spinal cord, brain stem, and diencephalon), which is responsible for targeting lower motor neurons in the spinal cord that are involved in reflexes, locomotion, complex movements, and postural control [59,62]. Repatterning also extends axonal linkage between neurons, facilitates the growth of neural nets, increases myelination, and facilitates new neural route pathways., as described by Sechenov [63,64] Pavlov [65], Anokhin [66], Haines [21], Virella [22].

The MNRI method addresses the neuro-sensorimotor aspect of early sensory-motor patterns and reflexes to support sensory-motor integration and neurodevelopment in children and adults with neuro deficits and learning challenges: CP, TBI [57,58,59,60], ASD [55] Down syndrome [55,56,67,68], and other neurological disorders [69,70,71]. MNRI method meets the ever-increasing demands for neurorehabilitation of individuals with impaired sensorimotor functions due to central nervous system damage or dysfunction. Previous studies of the MNRI Method and its different sub-programs demonstrated a positive effect on immune markers [56,72,73], neurophysiological functions [58,59], and various developmental aspects including the regulation of behavior and emotions, language, and communication [55,69,71,74].

### 4.2. Study Populatio

New England IRB approved this study (#: 20160464, Legacy IRB#: 15-466, Action Date: 02/11/2016; A WIRB Copernicus Group Company). All specialists leading the evaluations were certified by the NIH (National Institute of Health, Office of Extra Mutual Research) “Protecting Human Research Participants” in 2012-All participants were assigned codes to protect anonymity. Receipt of informed consent was received from all participants’ parents or legal guardians. MNRI Therapy Program was conducted and treatment administered by designated Specialists or MNRI Core Specialists who have completed the requirements for Continuing Professional Education in MNRI and clinical hours (www.MasgutovaMethod.com). Independent experts conducted a double-blind analysis of experimental results in the preparation of this report.

This study utilized a control group (*n* = 50) to provide a comparative analysis of neurotransmitters. The participants who provided samples for the neurotransmitter tests did not have inflammatory diseases, disorders, or allergies. The control group exclusion criteria included the following diagnosis: attention-deficit/hyperactivity disorder, anxiety, autism, and Asperger’s syndrome, Alzheimer’s disease, chronic migraines, depression, insomnia, obsessive-compulsive disorder, Parkinson’s disease, Cerebral Palsy, or PTSD. None of the individuals in the control group were taking medications or supplements at the time of sample submission. Only subjects aged 18 through 64 years were included, and a body mass index was calculated for all samples. Only individuals with BMI in the normal range normal (18.5–24.9), as defined by the U.S. Department of Health & Human Services, were included in the control group.

The study group (*n* = 116) contained participants who attended the MNRI conferences and signed the consent form, according to NEIRB regulations, did not add new supplements, medications, or participate simultaneously in other treatments, such as but not limited to Neurofeedback, CBD, or Cranial Sacral therapies. All participants were officially diagnosed and then categorized into five diagnosis groups. The participants were not financially compensated for the study participation. All samples were collected and processed according to the reference laboratory’s protocol. The samples that did not satisfy the acceptance or QC criteria were excluded from the study by the reference laboratory. The excluded samples were either too diluted or missed either post- or pre-sample values.

#### Description of Study Groups

Study group 1: Developmental Disorders (*n* = 34), including Developmental Delay, Dyslexia, Heat defects, restricted growth and development, Down syndrome, and general developmental delay.

Study group 2: Anxiety Disorders, OCD, PTSD (*n* = 20): subjects diagnosed, as per DSM-5 criteria, with disorders associated with anxiety, such as anxiety, obsessive-compulsive disorder, and post-traumatic stress disorder. Subjects with a major depressive mood disorder were not a part of this group.

Study group 3: Palsy and Seizures (*n* = 16) diagnosis included one or more neurological disorders such as cerebral palsy, seizures, or Tourettes.

Study group 4: ADD/ADHD (*n* = 24) all had an official diagnosis of attention deficit disorder or attention-deficit/hyperactivity disorder.

Study group 5: ASD (*n* = 22) inclusion of all diagnoses that fall within the Autism Spectrum Disorder according to The Diagnostic and Statistical Manual of Mental Disorders (DSM-5, published in 2013).

### 4.3. Laboratory Methods: Analysis of Urinary Catecholamine’s and Neurotransmitters

The urine test panel included nine neurotransmitters: epinephrine, norepinephrine, dopamine,4-dihydroxyphenylacetic acid (DOPAC), serotonin, 5-HIAA, glycine, glutamate, and histamine. The tested subjects were instructed to fast eight hours before going to bed but could drink plain water and take supplements or medications per doctors’ orders, or according to their six-month routine. On the morning of urine collection, the subjects fasted and did not drink any liquid. The first-morning urine was discarded; the second-morning urine was collected for analysis. All urinary samples were stored frozen at −20 °C and assayed within one week of collection. Urinary catecholamines and neurotransmitters were measured by competitive ELISA test. These IVD methods were CLIA approved and processed by a CLIA licensed reference laboratory (Pharmasan Labs, Osceola, WI, USA). All samples were collected and transported according to specifications provided by the reference laboratory. All analyses were blinded, without knowledge of subject diagnoses, current therapy, or targeted outcomes. This process was performed twice during the treatment on the first and the last day of the treatment. The course of treatment lasted 5 to 8 days, each day consisting of 6 h of MNRI treatment.

### 4.4. Statistical Analysis

All statistical analyses (calculation of mean, standard deviation, percent difference, statistical significance, and effect size) were calculated in Excel. A paired t-test was used to calculate the statistical significance (α = 0.05) between pre- and post-treatment of all disease groups and all parameters. The effect size for each parameter between groups (e.g., Group 1 and Group 2) was assessed by calculating Cohen’s *d*:

$$d=\frac{\mu_{1}\;-\;\mu_{2}}{\sqrt{\frac{\left(N_{1}\;-\;1\right)\sigma_{1}^{2}\;+\;\left(N_{2}\;-\;1\right)\sigma_{2}^{2}}{\left(N_{1}\;+\;N_{2}\;-\;2\right)}}},$$

where $N_{i}$ is the size of the group *i*, $\mu_{i}$ is the mean of an investigated parameter in group i, $\sigma_{i}^{2}$ is the variance of this parameter [13,75]. If an absolute value of *d* is between [0.2–0.5] the effect is small (Sm), [0.5–0.8] is a medium effect (Md), an absolute value between [0.8–1.2] indicates a large effect (Lg), and an absolute value greater than +1.2 is a very large effect (VL).

### 4.5. The MNRI Reflex Integration Therapy Modality

For those participants in the Study Group Experimental arm, each treatment session focused on a specific process of neurodevelopment. The MNRI Reflex Integration process included the following MNRI modules:

Reflex Repatterning—focuses on paving and improving the connectivity between the sensory and motor neurons in a reflex circuit [58,59] that influence the sensory-motor milestones, motor programming, planning and control, and cognitive skills [55,71,74].

NeuroStructural Reflex and Immune System Integration—focuses on improving the functions of reflexes responsible for postural control, spine flexibility, abdomen, neck, and limb musculature tone regulation, the release of core tendon guard creating positive protection and the feeling of being secure. Immunomodulatory effects include improvement of functions in T-1 immunity, cytokinesis, CD-4, CD-8, and other immune cell functions, anti-inflammatory effect, and regulation of immunoglobulins (IgE, IgG, and other) [56,67,68].

NeuroTactile Integration—focuses on the regulation and normalization of tactile sensitivity (hyper- or hypo-), coordination and integration of receptors, skin dermatomes, and overall peripheral and central nervous system for support of reflex repatterning and integration [74].

Archetype Movements Integration—focuses on the enhancement of the primary biomechanics of motor patterns (extension, flexion, rotation, stretching-compression, and other) giving support for structural aspect of numerous reflex patterns, development of automatic and consciously learned motor abilities and skills; also postural and motor control, with secondary improvement in the speed of perception, focusing, and memory, in sensory-motor integration, and cognitive functions [75].

Breathing Reflex Integration—focuses on the regulation and normalization of breathing reflex patterns, and the residual volume of the lungs for normal breathing and creating enough protection and survival [72].

Stress and Traumatic Stress Release—focuses on reflex patterns that can impact the HPA stress-axis for letting go of past negative stressors and traumas, and for trauma normalization by activation of stress hormone and neurotransmitter regulation [55,69].

Proprioceptive-Cognitive Integration—focuses on improving proprioceptive-vestibular (balance) system-related reflexes for support of postural and motor control, with secondary improvement in motor-cognitive functions.

Oral-Motor/Visual-Auditory Reflexes Integration—focuses on improving oral-motor, articulation, and speech abilities, as well as visual and auditory functions [69].

The fundamental goal of the MNRI module is to utilize reflex patterns for improvements of daily functioning in individuals with disruption of sensory-motor integration, increasing stress and immune system resilience, physical wellness, behavioral and emotional regulation, and cognitive skills. The typical duration of a Family Conference is eight days. The study participants receive six 50-min sessions of MNRI therapy programs daily.

## 5. Conclusions

We presented compelling evidence that MNRI therapy has multi-faceted influences on the neurological and endocrine systems. Physiological homeostasis, as well as immunological optimization, are impacted through hormone and neurotransmitter modulation. Results show that MNRI consistently reduces and works with mechanisms of reduction of inflammation in all five analyzed groups of disorders. We hypothesize that this physical-therapeutic, non-pharmaceutical approach is an intervention that not only reduces inflammation but also assists in the modulation of MAO and PMNT, therefore reducing oxidative stress, and encouraging homeostasis of excitatory neurotransmitters. Impacting oxidative stress, inflammation, and optimizing neurotransmitter levels will enhance neurological and endocrine health. Imbalances of neurotransmitters will influence mood, cognitive function, focus, behavior, motivation, and ability to assimilate or understand social norms. MNRI facilitates neurodevelopment, stress resiliency, neuroplasticity, and optimal learning opportunity. Note that every study group is traditionally treated with pharmacological or invasive medical treatments, having potentially severe side effects. There have been no reported side effects of MNRI treatments. Conclusions derived from the neurotransmitter test results have been corroborated by statements caregivers and participants themselves, reporting fewer social issues, fewer medical interventions needed, increased independence, and mood control.

In conclusion, we suggest that MNRI is a viable option for neurotransmitter regulation. Even a small study of a relatively short 8-day intervention demonstrated statistically and therapeutically significant results, therefore implying that continued treatment can achieve an even more substantial impact. Hence, we speculate the MNRI can be an efficient supplement to pharmaceutical and or surgical interventions or used as a stand-alone treatment. Further larger-scale studies are required to investigate the best intervention protocol.

## Figures and Tables

**Table 1:** Study Group Developmental disorders (*n* = 34). Comparisons between levels of epinephrine, norepinephrine, dopamine, serotonin, glutamate, glycine, histamine, DOPAC.

Developmental Disorders (*n* = 34)		Epinephrine (ug/g Cr)	Norepinephrine (ug/g Cr)	Glutamate (umol/g Cr)	Glycine (umol/g Cr)	bPEA (nmol/g Cr)	Histamine (ug/g Cr)	GABA (umol/g Cr)	DOPAC (umol/g Cr)	5-HIAA (ug/g Cr)
**Controls**	**Mean (μ)**	1.80	31.8	21.5	1004.8	40.7	24.7	4.21	468.9	2460.1
	**SD**	0.75	13.6	10.1	552.1	19.1	9.7	1.15	131.1	1434.8
**Pre-Tx**	**Mean (μ)**	4.64	62.3	70.9	1536.2	57.4	44.1	9.40	997.7	6271.0
	**SD**	2.91	27.7	49.9	558.7	18.3	20.1	3.43	744.7	3274.9
**Post-Tx**	**Mean (μ)**	3.46	62.2	65.7	1398.6	51.7	41.9	8.95	1003.5	6181.5
	**SD**	1.76	29.2	43.5	600.9	22.4	23.0	3.41	491.6	2788.6
**Diffe-rence (%)**	**Controls vs. PreTx**	158%	96%	229%	53%	41%	79%	123%	113%	155%
	**Controls vs. PostTx**	92%	95%	205%	39%	27%	70%	113%	114%	151%
	**PreTx vs. PostTx**	−25%	0%	−7%	−9%	−10%	−5%	−5%	1%	−1%
**Statis-tical Signi-ficance**	**Controls vs. PreTx**	***	***	***	**	**	***	***	***	***
	**Controls vs. PostTx**	***	***	***	*	T	***	***	***	***
	**PreTx vs. PostTx**	*	ns	ns	ns	ns	ns	ns	T	ns
**Effect Size**	**Controls vs. PreTx**	VL	VL	VL	Lg	Lg	VL	VL	Lg	VL
	**Controls vs. PostTx**	VL	VL	VL	Md	Md	Lg	VL	VL	VL
	**PreTx vs. PostTx**	Sm	NoEf	NoEf	Sm	Sm	NoEf	NoEf	NoEf	NoEf

5–HIAA for control (Ct), pre-treatment (Pr), and post-treatment (Ps). Asterixis *, **, and *** denote three levels of significance corresponding to *p*-value < 0.05, < 0.01, and < 0.001, respectively.

**Table 2:** Study Group Anxiety/OCD disorders (*n* = 20). Comparisons between levels of epinephrine, norepinephrine, dopamine, serotonin, glutamate, glycine, histamine, DOPAC.

Anxiety/OCD/ PTSD (*n* = 20)		Epinephrine (ug/g Cr)	Norepinephrine (ug/g Cr)	Glutamate (umol/g Cr)	Glycine (umol/g Cr)	bPEA (nmol/g Cr)	Histamine (ug/g Cr)	GABA (umol/g Cr)	DOPAC (umol/g Cr)	5-HIAA (ug/g Cr)
**Controls**	**Mean (μ)**	1.80	31.8	21.5	1004.8	40.7	24.7	4.21	468.9	2460.1
	**SD**	0.75	13.6	10.1	552.1	19.1	9.7	1.15	131.1	1434.8
**Pre-Tx**	**Mean (μ)**	3.05	50.5	65.3	1474.3	62.2	42.4	9.32	714.8	6385.2
	**SD**	1.41	20.5	42.8	404.6	33.1	21.4	4.01	233.6	3368.1
**Post-Tx**	**Mean (μ)**	2.98	50.3	53.2	1367.1	55.8	41.0	8.40	796.5	6195.0
	**SD**	1.29	27.5	30.3	442.9	26.0	21.0	3.63	242.2	2886.2
**Difference (%)**	**Controls vs. PreTx**	70%	59%	203%	47%	53%	72%	121%	52%	160%
	**Controls vs. PostTx**	66%	58%	147%	36%	37%	66%	99%	70%	152%
	**PreTx vs. PostTx**	−2%	0%	−19%	−7%	−10%	−3%	−10%	11%	−3%
**Statistical Signi-ficance**	**Controls vs. PreTx**	***	***	***	*	*	***	***	***	***
	**Controls vs. PostTx**	***	**	***	T	*	***	***	***	***
	**PreTx vs. PostTx**	ns	ns	ns	ns	ns	ns	ns	ns	ns
**Effect Size**	**Controls vs. PreTx**	Lg	Lg	VL	Lg	Md	Lg	VL	VL	VL
	**Controls vs. PostTx**	Lg	Lg	VL	Md	Md	Lg	VL	VL	VL
	**PreTx vs. PostTx**	NoEf	NoEf	Sm	Sm	Sm	NoEf	Sm	Sm	NoEf

5–HIAA for control (Ct), pre-treatment (Pr), and post-treatment (Ps). Asterixis *, **, and *** denote three levels of significance corresponding to *p*-value < 0.05, <0.01, and <0.001, respectively.

**Table 3:** Study Group Palsy & Seizures disorders (*n* = 16). Comparisons between levels of epinephrine, norepinephrine, dopamine, serotonin, glutamate, glycine, histamine, DOPAC.

Palsy & Seizures (*n* = 16)		Epinephrine (ug/g Cr)	Norepinephrine (ug/g Cr)	Glutamate (umol/g Cr)	Glycine (umol/g Cr)	bPEA (nmol/g Cr)	Histamine (ug/g Cr)	GABA (umol/g Cr)	DOPAC (umol/g Cr)	5-HIAA (ug/g Cr)
**Controls**	**Mean (μ)**	1.80	31.8	21.5	1004.8	40.7	24.7	4.21	468.9	2460.1
	**SD**	0.75	13.6	10.1	552.1	19.1	9.7	1.15	131.1	1434.8
**Pre-Tx**	**Mean (μ)**	4.21	59.0	101.9	3136.9	76.2	52.9	15.01	921.9	10119.4
	**SD**	2.61	20.6	49.2	1681.4	36.2	20.7	7.29	544.5	5422.2
**Post-Tx**	**Mean (μ)**	3.45	66.6	103.9	2734.5	80.5	52.8	13.48	878.0	10331.0
	**SD**	1.70	24.7	41.8	1312.0	39.3	25.8	6.96	379.7	6384.1
**Difference (%)**	**Controls vs. PreTx**	134%	85%	373%	212%	87%	114%	257%	97%	311%
	**Controls vs. PostTx**	92%	109%	382%	172%	98%	114%	220%	87%	320%
	**PreTx vs. PostTx**	−18%	13%	2%	−13%	6%	0%	−10%	−5%	2%
**Statis-tical Signi-ficance**	**Controls vs. PreTx**	***	***	***	***	***	***	***	***	***
	**Controls vs. PostTx**	***	***	***	***	***	***	***	***	***
	**PreTx vs. PostTx**	ns	ns	ns	ns	ns	ns	ns	ns	ns
**Effect Size**	**Controls vs. PreTx**	VL	VL	Hg	VL	VL	VL	Hg	Lg	VL
	**Controls vs. PostTx**	VL	VL	Hg	VL	VL	VL	VL	VL	VL
	**PreTx vs. PostTx**	Sm	Sm	NoEf	Sm	NoEf	NoEf	Sm	NoEf	NoEf

5–HIAA for control (Ct), pre-treatment (Pr), and post-treatment (Ps). Asterixis *, **, and *** denote three levels of significance corresponding to *p*-value < 0.05, < 0.01, and < 0.001, respectively.

**Table 4:** Study Group ADD/ADHD disorders (*n* = 24). Comparisons between levels of epinephrine, norepinephrine, dopamine, serotonin, glutamate, glycine, histamine, DOPAC.

ADD/ADHD (*n* = 24)		Epinephrine (ug/g Cr)	Norepinephrine (ug/g Cr)	Glutamate (umol/g Cr)	Glycine (umol/g Cr)	bPEA (nmol/g Cr)	Histamine (ug/g Cr)	GABA (umol/g Cr)	DOPAC (umol/g Cr)	5-HIAA (ug/g Cr)
**Controls**	**Mean (μ)**	1.80	31.8	21.5	1004.8	40.7	24.7	4.21	468.9	2460.1
	**SD**	0.75	13.6	10.1	552.1	19.1	9.7	1.15	131.1	1434.8
**Pre-Tx**	**Mean (μ)**	3.04	60.6	74.6	1541.7	68.7	49.5	8.88	678.1	5527.5
	**SD**	1.47	28.4	50.2	582.0	32.9	22.5	3.53	211.5	2569.4
**Post-Tx**	**Mean (μ)**	2.87	57.7	55.3	1332.4	54.6	39.3	7.71	845.0	5327.7
	**SD**	1.28	30.8	33.4	433.0	25.0	19.6	3.12	307.6	2020.1
**Diffe-rence (%)**	**Controls vs. PreTx**	69%	90%	246%	53%	69%	101%	111%	45%	125%
	**Controls vs. PostTx**	59%	81%	157%	33%	34%	59%	83%	80%	117%
	**PreTx vs. PostTx**	−6%	−5%	−26%	−14%	−21%	−21%	−13%	25%	−4%
**Statis-tical Significance**	**Controls vs. PreTx**	***	***	***	**	***	***	***	***	***
	**Controls vs. PostTx**	***	***	***	T	*	***	***	***	***
	**PreTx vs. PostTx**	ns	ns	*	ns	*	*	T	**	ns
**Effect Size**	**Controls vs. PreTx**	Lg	VL	VL	Lg	Lg	VL	VL	Lg	VL
	**Controls vs. PostTx**	Lg	Lg	VL	Md	Md	Lg	VL	VL	VL
	**PreTx vs. PostTx**	NoEf	NoEf	Sm	Sm	Sm	Sm	Sm	Md	NoEf

5–HIAA for control (Ct), pre-treatment (Pr), and post-treatment (Ps). Asterixis *, **, and *** denote three levels of significance corresponding to *p*-value < 0.05, < 0.01, and < 0.001, respectively.

**Table 5:** Study Group Autism spectrum disorders (*n* = 22). Comparisons between levels of epinephrine, norepinephrine, dopamine, serotonin, glutamate, glycine, histamine, DOPAC.

Autism Spectrum Disorder (*n* = 22)		Epinephrine (ug/g Cr)	Norepinephrine (ug/g Cr)	Glutamate (umol/g Cr)	Glycine (umol/g Cr)	bPEA (nmol/g Cr)	Histamine (ug/g Cr)	GABA (umol/g Cr)	DOPAC (umol/g Cr)	5-HIAA (ug/g Cr)
**Controls**	**Mean (μ)**	1.80	31.8	21.5	1004.8	40.7	24.7	4.21	468.9	2460.1
	**SD**	0.75	13.6	10.1	552.1	19.1	9.7	1.15	131.1	1434.8
**Pre-Tx**	**Mean (μ)**	6.64	55.6	55.5	1640.1	71.7	55.5	9.13	730.5	7142.1
	**SD**	6.08	26.4	34.2	585.4	34.1	32.3	3.03	339.7	3173.6
**Post-Tx**	**Mean (μ)**	6.57	46.2	54.7	1584.6	60.6	48.4	7.69	750.6	7240.0
	**SD**	6.20	24.0	35.8	741.9	35.1	32.4	2.29	296.7	2763.0
**Diffe-rence (%)**	**Controls vs. PreTx**	269%	75%	158%	63%	76%	125%	117%	56%	190%
	**Controls vs. PostTx**	265%	45%	154%	58%	49%	96%	83%	60%	194%
	**PreTx vs. PostTx**	−1%	−17%	−2%	−3%	−16%	−13%	−16%	3%	1%
**Statis-tical Signi-ficance**	**Controls vs. PreTx**	***	***	***	***	***	***	***	***	***
	**Controls vs. PostTx**	***	**	***	**	**	***	***	***	***
	**PreTx vs. PostTx**	ns	*	ns	ns	T	ns	*	ns	ns
**Effect Size**	**Controls vs. PreTx**	Lg	Lg	VL	Lg	Lg	VL	Hg	Lg	VL
	**Controls vs. PostTx**	Lg	Md	VL	Lg	Md	Lg	VL	VL	Hg
	**PreTx vs. PostTx**	NoEf	Sm	NoEf	NoEf	Sm	Sm	Md	NoEf	NoEf

5–HIAA for control (Ct), pre-treatment (Pr), and post-treatment (Ps). Asterixis *, **, and *** denote three levels of significance corresponding to *p*-value < 0.05, < 0.01, and < 0.001, respectively.

## Abbreviations

MNRI	Masgutova Neurosensorimotor Reflex Neuromodulation Intervention
ADHD	Attention-Deficit/Hyperactivity Disorder
OCD	Obsessive-Compulsive Disorder
TAAR1	The Trace Amine-Associated Receptor
MAO	Monoamine Oxidase A
PMNT	Phenylethanolamine N-Methyltransferase
CNS	Central Nervous System
SNS	Sympathetic Nervous System
PNS	Parasympathetic Nervous System
PEA	Palmitoylethanolamide
ASD	Autism Spectrum Disorder
HPA	Hypothalamus-Pituitary-Adrenal
ADD	Attention Deficit Disorder
NMDA	N-Methyl-D-Aspartate Receptor
